# Neurofeedback-Augmented Mindfulness Training Elicits Distinct Responses in the Subregions of the Insular Cortex in Healthy Adolescents

**DOI:** 10.3390/brainsci12030363

**Published:** 2022-03-09

**Authors:** Xiaoqian Yu, Zsofia P. Cohen, Aki Tsuchiyagaito, Gabriella Cochran, Robin L. Aupperle, Jennifer L. Stewart, Manpreet K. Singh, Masaya Misaki, Jerzy Bodurka, Martin P. Paulus, Namik Kirlic

**Affiliations:** 1 Laureate Institute for Brain Research, Tulsa, OK 74136, USA; xyu@laureateinstitute.org (X.Y.); zcohen@laureateinstitute.org (Z.P.C.); atsuchiyagaito@laureateinstitute.org (A.T.); gcochran@laureateinstitute.org (G.C.); raupperle@laureateinstitute.org (R.L.A.); jstewart@laureateinstitute.org (J.L.S.); mmisaki@laureateinstitute.org (M.M.); mpaulus@laureateinstitute.org (M.P.P.); 2 Department of Community Medicine, University of Tulsa, Tulsa, OK 74104, USA; 3 Department of Psychiatry and Behavioral Sciences, Stanford University, Stanford, CA 94305, USA; mksingh@stanford.edu

**Keywords:** mindfulness, interoception, insula, real-time fMRI neurofeedback, awareness, adolescents

## Abstract

Mindfulness training (MT) reduces self-referential processing and promotes interoception, the perception of sensations from inside the body, by increasing one’s awareness of and regulating responses to them. The posterior cingulate cortex (PCC) and the insular cortex (INS) are considered hubs for self-referential processing and interoception, respectively. Although MT has been consistently found to decrease PCC, little is known about how MT relates to INS activity. Understanding links between mindfulness and interoception may be particularly important for informing mental health in adolescence, when neuroplasticity and emergence of psychopathology are heightened. We examined INS activity during real-time functional magnetic resonance imaging neurofeedback-augmented mindfulness training (NAMT) targeting the PCC. Healthy adolescents (N = 37; 16 female) completed the NAMT task, including Focus-on-Breath (MT), Describe (self-referential processing), and Rest conditions, across three neurofeedback runs and two non-neurofeedback runs (Observe, Transfer). Regression coefficients estimated from the generalized linear model were extracted from three INS subregions: anterior (aINS), mid (mINS), and posterior (pINS). Mixed model analyses revealed the main effect of run for Focus-on-Breath vs. Describe contrast in aINS [R^2^ = 0.39] and pINS [R^2^ = 0.33], but not mINS [R^2^ = 0.34]. Post hoc analyses revealed greater aINS activity and reduced pINS activity during neurofeedback runs, and such activities were related to lower self-reported life satisfaction and less pain behavior, respectively. These findings revealed the specific involvement of insula subregions in rtfMRI-nf MT.

## 1. Introduction

Mindfulness refers to the moment-to-moment, non-judgmental awareness that is cultivated by paying attention in a particular way: on purpose, in the present moment, and non-reactively [1]. Dispositional mindfulness appears to be positively related to psychological health [2,3], and mindfulness training (MT) has been shown to enhance psychological well-being in healthy populations [4,5], as well as benefit various health conditions including chronic pain, stress, anxiety, depression, addiction, and suicidality [6,7,8]. Adolescence represents a sensitive developmental period for emergence of psychopathology, including anxiety disorders [9], depression [10], and suicidal behavior [11]. Although early interventions such as school-based MT have demonstrated effectiveness in enhancing well-being in healthy adolescents [12,13], there is still a need to delineate the precise mechanisms by which MT exerts its mental health benefits to optimize prevention and early intervention efforts in youth.

### 1.1. The Role of Interoception in Mindfulness and Its Key Hub Insula

By focusing attention on the body and on the “here and now”, MT directly cultivates interoception [14,15,16,17], the individual’s ability to focus attention on the current body state to select and motivate most appropriate regulatory behaviors [18]. In fact, interoception has been proposed as one of the core mechanisms of action of MT [17,19,20,21]. MT ranging from 3 to 12 weeks has been found to improve interoceptive awareness as measured by the self-reported Multidimensional Assessment of Interoceptive Awareness (MAIA) in various clinical populations, including patients with chronic pain and comorbid depression, who reported greater increase in the self-regulation and non-distracting subscales after the 8 week Mindfulness-Based Cognitive Therapy (MBCT) [16]; veterans with post-traumatic stress disorder (PTSD) who reported increases in the self-regulation, body listening, and emotional awareness subscales after a 12 week integrative exercise program [22]; and depressed patients who showed improved regulatory and belief-related aspects of interoception as measured by the attention regulation, self-regulation, body listening, and trusting subscales after a 3 week MBCT [23].

The insular cortex (INS) has been identified as the key hub for interoception [24,25,26,27,28]. Characterizing increased complexity in INS functioning, INS can be subdivided into three subregions from posterior to anterior orientation (i.e., posterior-to-anterior progression hypothesis) [25,29]. Specifically, the posterior INS (pINS) is primarily involved in encoding interoceptive signals, the mid-INS (mINS) is important in integrating interoception with motivated behavior, and the anterior INS (aINS) encodes both introspective emotional awareness and bodily sensations [26,30]. Although several neuroimaging studies have examined the relation between MT and INS activity, the majority of them have reported INS activity as a whole, while extant findings are largely mixed. Earlier research suggested that MT reduced self-referential processing that was accompanied by decreased aINS activations during meditation among experienced meditators [31]. However, others have proposed that INS would show increased activation due to the stronger interoception that is cultivated through MT [19]. Indeed, a review of nine neuroimaging studies found increased INS activity following either the eight-week Mindfulness-Based Stress Reduction (MBSR) course [32] or mindfulness-based tasks among stressed, anxious, and healthy participants [33]. The majority of the literature evaluating insula subregions has reported effects on aINS, rather than mINS or pINS [34]. The mixed INS findings may relate to there being functionally distinct INS subregions involved in affective relative to somatosensory processing or may be explained by unaccounted individual, measurement, or treatment differences. Nevertheless, mixed findings and limited empirical data linking MT and activity in INS subregions require further clarification of the role of INS and interoception in MT.

### 1.2. Real-Time Functional Magnetic Resonance Imaging Neurofeedback-Augmented MT Targeting the Posterior Cingulate Cortex

The use of real-time functional magnetic resonance imaging neurofeedback (rtfMRI-nf) provides the means of experimentally testing and modulating brain networks central to MT, thereby allowing for a more direct assessment of the role INS plays in this process. MT engages distributed network of brain regions, particularly the regions of the default mode network (DMN) [35,36,37,38], whose central hub is the posterior cingulate cortex (PCC) that supports self-referential processing, the cognitive process of relating information to the self [39]. Indeed, changes in the PCC are among the most robust findings in mindfulness studies, as demonstrated by a recent review where near half of 49 neuroimaging studies reported significant effects of MT on the PCC [40]. PCC has been shown to be activated during mind wandering and self-referential processing [41,42], and deactivated during various mindfulness/meditation practices [43]. Importantly, both INS and PCC play interconnected roles in MT that PCC is also known to be involved in self-awareness [44,45], a construct is often used to measure self-reported interoception (i.e., the Self-Awareness Questionnaire [46] and the How do you feel questionnaire [47]). Given that PCC may be the key modulatory target of MT, neurofeedback targeting the PCC during MT has been successfully implemented in adults [48] and in our recent work with adolescents [49]. Specifically, we report the feasibility and tolerability of PCC-targeted rtfMRI-nf-augmented mindfulness training (MT) in the current sample as established by self-report and successful downregulation of PCC as a function of rtfMRI-nf-augmented mindfulness training and above and beyond mindfulness training alone. In addition, the whole-brain and functional connectivity analyses found co-modulation of a range of regions in the default mode and salience networks, including with the pINS.

### 1.3. The Present Study

Based on previous literature and our findings pointing to co-modulation of the pINS consequent to rtfMRI-nf targeting the PCC during mindfulness training [49], the present study aimed to clarify the role of INS in MT. We examined activity in distinct INS subregions during MT and its modulation with rtfMRI-nf-augmented MT (NAMT) targeting PCC in healthy adolescents. We selected this developmental period because adolescent brain neuroplasticity allows for effective attempts at improving learning and performance, and therefore makes it a critical period to study neural correlates of psychological and behavioral strategies and their optimization [50,51]. Because aINS is better studied than mINS and pINS in mindfulness research [34], the primary outcome was aINS activity during Focus-on-Breath (i.e., MT) relative to Describe (i.e., self-referential processing). The secondary outcomes included mINS and pINS activity under the same condition. Given that insula activation has been found to be the most consistent effect observed following MT in meditation-naïve participants [34], we hypothesized that (H1) relative to self-referential processing, aINS would show increased activity during MT in healthy adolescents, and (H2): this activation will be further modulated consequent to rtfMRI-nf. Our approach regarding the activity in mINS and pINS in relation to MT was exploratory given the limited research in this area [34]. For exploratory outcomes, we collected self-reported measures concerning task adherence (task ratings) measured immediately following each task run, the State Mindfulness Scale (SMS) measured pre- and post-NAMT [52], as well as affective and sensory awareness assessed by the Patient-Reported Outcomes Measurement Information System (PROMIS) Pediatric scales measured pre-NAMT, including life satisfaction [53], meaning and purpose [54], positive affect [55], pain behavior [56], pain interference [57], and fatigue [58]. Based on the posterior-to-anterior progression hypothesis [25,29] and awareness of emotion and sensory states being core to interoception and mindfulness [14,59], we hypothesized that aINS activity would be related to affective awareness (i.e., life satisfaction, meaning and purpose, and positive affect), while mINS and pINS activity would be related to sensory awareness such as pain behavior and pain interference (H3).

## 2. Materials and Methods

### 2.1. Participants

Adolescents in the present study took part in a larger ongoing longitudinal study, with recruitment taking place between September 2019 and July 2021. Adolescents were recruited from the community using flyers, radio and social media advertisements, billboards, and a school-based messaging platform (i.e., PeachJar). A phone screen determined initial eligibility. Remote and in-person visits with adolescents and primary caregivers provided demographic information, medical and psychiatry history, pubertal status, family history of psychiatric illness, and an MRI safety questionnaire. Eligible adolescents were between 13 and 17 years of age at the time of enrollment, had a parent or a legal guardian able to provide consent, were psychiatrically and physically healthy, and were able to validly and safely complete baseline assessments. All races and genders were included. Adolescents were excluded if diagnosed with a neurological or developmental disorder, were currently being managed for migraines (e.g., daily prophylactic medication), had history of traumatic brain injury, had a lifetime history of psychopathology, were currently using medications with major effects on brain function or blood flow (e.g., acne medication), and/or reported MRI contraindications. Forty adolescents were consented for the present study, with two adolescents withdrawn due to repeated missed appointments following consent procedures, and one participant not having usable data due to technical difficulties, for a total of 37 healthy adolescents included in current analyses. According to Desmond and Glover [60], a sample size of 24 is recommended for typical within-group fMRI experiments, in which inferences regarding the differences in activation between two or more conditions are intended to be made in a single population. Therefore, with N = 37, we were 98% powered to detect medium-size effects (*f* = 0.25) between conditions. Thirty-four subjects overlapped with the sample in [49]. 

### 2.2. Experimental Procedures

This study involves analysis of an ongoing study focused on the impact of self-regulation of PCC using rtfMRI-nf. The current study presents a tangential analysis to the primary outcomes as delineated on Clinical Trials (www.clinicaltrials.gov NCT04053582).

#### 2.2.1. Neurofeedback-Augmented Mindfulness Training Task (NAMT)

**Figure 1:**
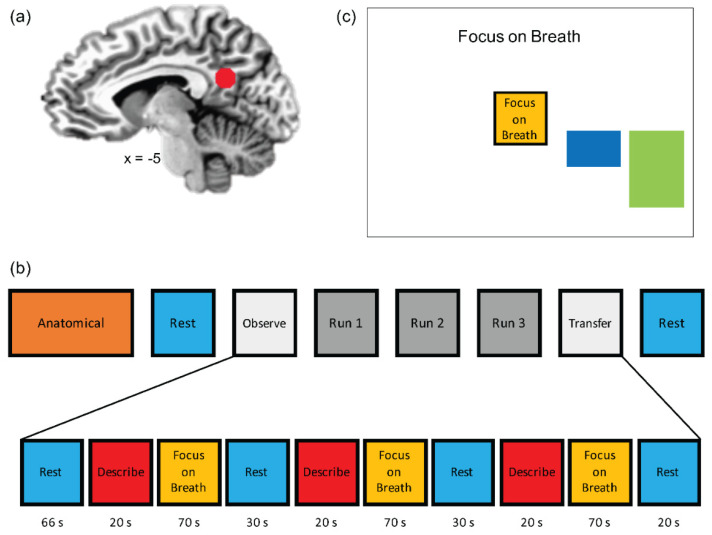
Real-time fMRI neurofeedback-augmented mindfulness training task [49]. (**a**) Posterior cingulate cortex (PCC, MNI coordinates: x = −5, y = −55, z = 23) was selected as the target (ROI, spheres of 7 mm radius) for the real-time fMRI neurofeedback (rtfMRI-nf) training. (**b**) The experimental protocol consisted of eight fMRI runs, including an anatomical scan, Resting State scan 1 (Rest-1), Observe (OBS), three neurofeedback runs (NF-1, NF-2, NF-3), a Transfer run (TRS), and Resting State scan 2 (Rest-2). During Rest runs (lasting 6 min), the participants were instructed to clear their minds and not to think about anything in particular while fixating at the display screen. OBS, NF-1, NF-2, NF-3, and TRS runs each lasted 6 min and 56 s. They started with a 66 s rest block, followed by alternating Focus-on-Breath (mindfulness training condition; 70 s), Describe (Active baseline condition; 20 s), and Rest (Baseline condition; 30 s) blocks. During the Focus condition, participants were instructed to pay attention to the physical sensations of their breath, not trying to change it in any way, and if their attention were to wander to something else, to gently bring it back to their breath. In the Describe condition, participants were presented with various adjectives, which they had to mentally categorize as descriptive or not descriptive of them. During the Rest condition, the participants were presented with the cue “Rest” and asked to relax while looking at the display screen. No neurofeedback was provided (no bars displayed) during the Rest and Describe conditions or during the entire OBS and TR runs. (**c**) During the Focus condition, participants viewed a graphical user interface (GUI) screen with neurofeedback bars (blue) and target bars (green). The participants were told that the blue bar may change with their experience of focusing on the breath, and that their goal was to make the blue bar match the green bar as often as possible. The target levels were −0.5%, −0.75%, and −1.0% (% signal change relative to the preceding rest block) for NF-1, NF-2, and NF-3, respectively.

The NAMT task (Figure 1) has been previously described [49], and further task details can be found in Appendix A. Briefly, adolescents were first given a brief psychoeducational introduction into mindfulness, followed by a guided traditional mindfulness practice focused on the breath [43,48]. Next, adolescents completed the same mindfulness practice and assessment with MRI noises in the background in the mock scanner. MT was manualized to ensure fidelity across participants. MT was delivered by a trained research assistant under the supervision of a licensed clinical psychologist. Training sessions were audio recorded and up to 20% sessions were randomly selected for fidelity ratings by research staff using an unpublished measure developed by NK in consultation with RLA for the purposes of this study. On 3-point Likert scale (0 = no adherence, 1 = adherence identified but weak or flawed, 2 = good adherence) of how closely the research assistant followed the manualized mindfulness training, the fidelity ratings indicate that the manualized training was delivered with satisfactory adherence (M = 95.81%, SD = 3.33%) (the percentage was obtained by summed score/highest total score possible).

The neuroimaging session included 8 runs (Figure 1a), including an anatomical scan, Resting State scan 1 (Rest-1), Observe (OBS), three neurofeedback runs (NF-1, NF-2, NF-3), Transfer run (TRS), and Resting State scan 2 (Rest-2). During Rest-1 and Rest-2 (6 min each), participants were instructed to clear their mind and not think about anything while fixating upon a fixation cross. OBS, NF-1, NF-2, NF-3, and TRS runs each lasted 6 min and 56 s. Runs started with a 66 s rest block, followed by alternating Describe (Active Control condition without neurofeedback; 20 s), Focus-on-Breath (MT condition with PCC neurofeedback; 70 s), and Rest (Baseline condition; 30 s) blocks. OBS and TRS runs did not involve neurofeedback (no bar displayed) during the Focus-on-Breath condition. During the Focus-on-Breath condition [48], adolescents were instructed to pay attention to the physical sensations of their breath, not trying to change their breathing in any way, and if their attention were to wander to something else, to gently bring their attention back to their breath [43]. In the Describe condition, adolescents were presented with various adjectives, which they had to mentally categorize as descriptive or not descriptive of them for the entire duration the word was displayed on the screen [61]. During neurofeedback runs, adolescents were told that they would see a bar displayed on the screen, representing the relative brain activity in a particular brain region in real time (Figure 1b). The instructions further indicated that the bar may change with the experience of focusing on the breath (i.e., the bar may go blue if they are fully concentrating on their breath, and red if their mind wanders elsewhere). The consensus on the reporting and experimental design of clinical and cognitive-behavioral neurofeedback studies (CRED-nf checklist) [62] is included in Appendix B Table A1.

#### 2.2.2. Psychological Measurements

Following each fMRI run, participants completed task ratings, including the following items: “How much did your mind wander while you were asked to focus on your breath?”, “How easy did you find it to focus on your breath?”, “How do you feel right now?”, each rated from 1 to 10. Participants completed the SMS at T1 (pre-) and T2 (post-NAMT) to quantify adolescents’ perceived level of attention to and awareness of their present experience (i.e., mind, body, the pleasant/unpleasant/neutral hedonic tones of these objects of awareness, and the qualities thought to characterize mindful awareness) [52]. Additionally, adolescents completed the following PROMIS Pediatric scales pre-NAMT: life satisfaction (example items: Thinking about the past 4 weeks, “I was satisfied with my life”, “I had a good life”, 1—Not at all, 5—Very much) [53], meaning and purpose (example items: Thinking about my life “I can reach my goals in life”, “I have a reason for living”, 1—Not at all, 5—Very much) [54], positive affect (example items: In the past 7 days, “I felt happy”, “I felt peaceful”,1—Never, 5—Always) [55], pain behavior (example items: In the last 7 days, when I was in pain, “It showed on my face”, “I talked about my pain.” 1—Never, 5—Always) [56], pain interference (example items: In the past 7 days, “I had trouble sleeping when I had pain”, “I felt angry when I had pain”, 1—Not at all, 5—Very much) [57], and fatigue (example items: In the past 7 days, “How often did you have to push yourself to get things done because of your fatigue?”, “I have trouble starting things because I am tired”, 1—Not at all, 5—Very much) [58]. 

#### 2.2.3. Data Acquisition

Neuroimaging was performed using a GE MR750 3T MRI scanner with the 8-channel receive-only head coil. To acquire T1-weighted anatomical images, a 3D magnetization-prepared rapid gradient echo (MPRAGE) pulse sequence accelerated with sensitivity encoding (SENSE) [63] was used. The MPRAGE parameters were as follows: FOV/slice thickness = 240/1.2 mm, axial slices per slab = 128, image matrix size = 256 × 256, TR/TE = 5.0/1.9 ms, SENSE acceleration factor R = 2, flip angle = 8°, delay/inversion times TD/TI = 1400/725 ms, sampling band- width = 31.2 kHz, and scan time = 5 min 33 s. 

For the whole-brain fMRI recording, an accelerated single-shot gradient EPI with SENSE was used. EPI sequence parameters were optimized to maximize sensitivity to BOLD contrast and minimalize image distortion and susceptibility dropouts [64,65]. EPI parameters were as follows: FOV/slice = 240/2.9 mm, TR/TE = 2000/25 ms, SENSE acceleration R = 2, acquisition matrix: 96 × 96, flip angle = 90°, image matrix: 128 × 128, 46 axial slices, and voxel volume: 1.9 × 1.9 × 2.9 mm^3^. To allow the fMRI signal to reach a steady state, three EPI volumes (6 s) were added at the beginning of each run and were excluded from data analysis. Physiological pulse oximetry and respiration waveforms were recorded simultaneously with fMRI (with 25 ms sampling interval, i.e., the sampling rate of the pulse oximeter and respiration measurements) using a photoplethysmograph placed on the subject’s finger and a pneumatic respiration belt, respectively. rtfMRI-nf procedures are described elsewhere [49] and in Appendix A.

#### 2.2.4. Data Processing and Analysis

AFNI [66] was used for data image analysis. The first 5 fMRI volumes were discarded to wait for a steady state. fMRI data preprocessing included despiking, RETROICOR [67], respiration volume per time correction [68], slice-timing and motion corrections, non-linear warping to the Montreal Neurological Institute (MNI) template brain with resampling to 2 mm^3^ voxels using the ANTs [69], spatial smoothing with a 6 mm FWHM Gaussian kernel, and scaling signal to percent change relative to the mean in each voxel. The general linear model (GLM) analysis was used for independently evaluating the brain response in the OBS, NF-1, NF-2, NF-3, and TRS runs. One participant included in analysis had no NF-2 due to technical difficulties. The design matrix included a modeled response to the Focus-on-Breath block (boxcar function convolved with hemodynamic response function), 12 motion parameters (3 shift and 3 rotation parameters with their temporal derivatives), three principal components of the ventricle signal, local white matter average signal (ANATICOR) [70], and low-frequency fluctuation (fourth-order Legendre polynomial model). 

**Figure A1:**
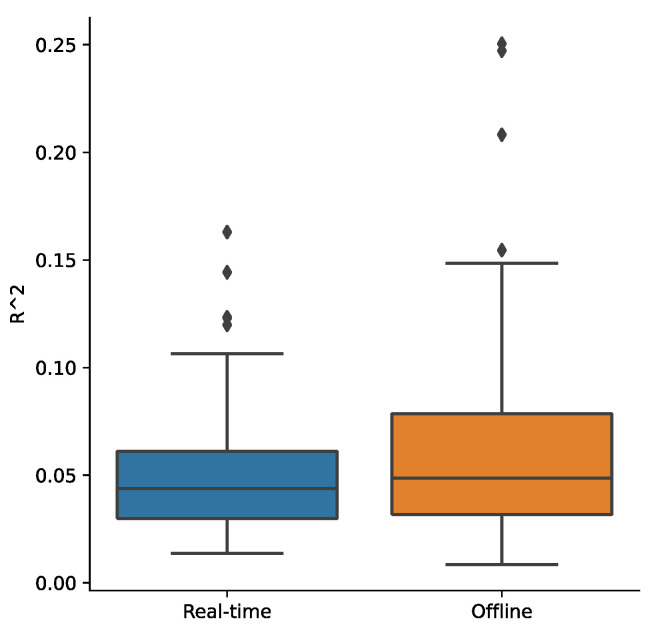
Barplot of the signal variance ratio (R^2 value) explained by the physiological noise regressors (RETROICOR) for the real-time processed and offline processed signals in the PCC region.

Our study is one of the first to include real-time physiological noise correction, and then offline RETROICOR correction in our preprocessing pipeline was followed by further physiological noise correction during the subsequent GLM analysis. Such a sequential approach of artifact removal can potentially reintroduce previously removed noise artifacts in later steps [71], thus it is important to evaluate the efficacy of physiological noise correction. We calculated the signal variance ratio (R^2^ value) explained by the physiological noise regressors (RETROICOR) for the real-time processed and offline processed signals in the PCC region. This measure indicates an amount of residual physiological noise effect in the processed signal [72]. The R^2^ was small for both real-time and offline-processed signals on average (Appendix B Figure A1). While some subjects’ runs showed a relatively high residual noise variance ratio, there was no significant correlation between the mean neurofeedback signal amplitude and the residual noise variance ratio for the real-time processed data (Spearman’s rho = −0.091, *p* = 0.361). Although a relatively high residual physiological noise variance ratio was seen for the offline-processed data, the correlation between the PCC parameter estimates (beta value) and the physiological noise variance ratio was not significant for the offline-processed data (Spearman’s rho = 0.031, *p* = 0.744). These indicate that the physiological noise effect was not significant on the neurofeedback signal in the real-time analysis and the PCC parameter estimation in the offline analysis.

**Figure 2:**
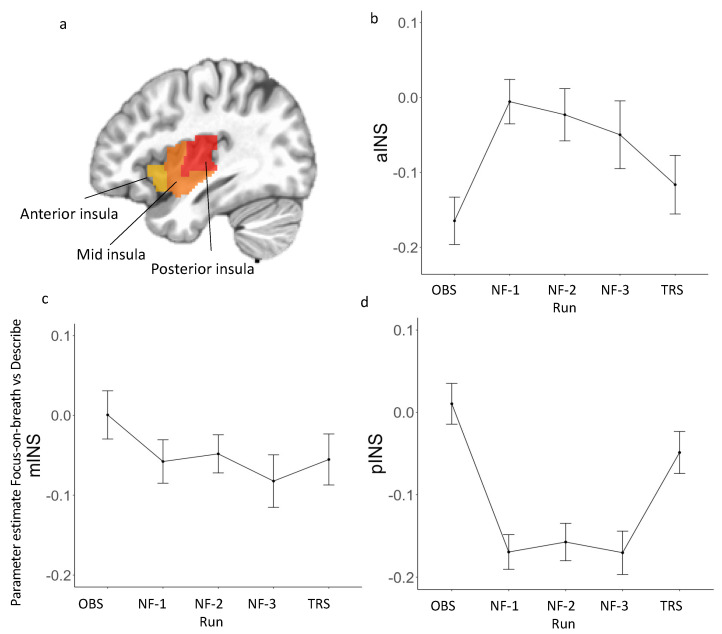
(**a**) Three insular cortex regions of interest (ROIs) extracted from the Brainnetome Atlas. (**b**–**d**) Parameter estimate (Focus-on-Breath vs. Describe) across each run in each insula subregion. See Appendix B Table A2 for peak coordinates for each insula subregion. Abbreviations: OBS, Observe; NF, Neurofeedback; TRS, Transfer.

Regression coefficients estimated from the GLM were extracted from both hemispheres of all three probabilistic cytoarchitectonic segmentations of INS regions defined by the Brainnetome atlas [73]: aINS (ventral and dorsal agranular), mINS (ventral dysgranular and granular, and dorsal dysgranular), and pINS (hypergranular and dorsal granular) averaged across left and right hemispheres (Figure 2a). See Appendix B Table A2 for peak coordinates of each subregion. The average parameter estimate (beta coefficient) of the Focus-on-Breath vs. Describe block regressor was extracted to evaluate brain activation during each run (OBS, NF-1, NF-2, NF-3, and TRS; H1). 

All remaining statistical analyses were performed using the R statistical package [74]. Descriptive statistics regarding participant characteristics and PROMIS pediatric measures were obtained using the R package ‘psych’ [75]. To test H2, first, Pearson’s correlation was conducted to examine the relation between parameter estimate (Focus-on Breath vs. Describe) in PCC and INS subregions; then separate linear mixed-effects models (LMEs) were conducted to examine task ratings and INS subregions activity across runs (OBS, NF-1, NF-2, NF-3, and TRS) using the ‘lmer’ function in the R package ‘lme4′ [76], with Run entered as a fixed effect and subjects as a random effect. Regarding exploratory outcomes (H3), LME was conducted for SMS, where Time (T1: pre-, T2: post-NAMT) was entered as a fixed effect and subjects as a random effect. Follow-up pairwise comparisons for LMEs were conducted using the ‘glht’ function in R package [77] and corrected for multiple comparisons with Tukey’s Honestly Significant Difference test. Spearman’s correlation analysis was conducted to examine the relation between parameter estimate (Focus-on Breath vs. Describe) in each INS subregion during neurofeedback runs (averaged across NF-1, NF-2, NF-3) and PROMIS pediatric measures. Bonferroni correction was used to correct for multiple correlation comparisons.

#### 2.2.5. Data and Code Availability Statement

The data and data analysis scripts that support the findings of this study are available on request from the corresponding author after a formal data sharing agreement has been signed. The data are not publicly available due to privacy or ethical restrictions.

## 3. Results

### 3.1. Demographic, Task, and Clinical Characteristics

Participants were 37 adolescents (age 14.61 ± 1.25 years, 16 female), and the majority were White (71%). Table 1 provides additional demographic information. LME results of self-report data are in Table 2. In general, adolescents reported moderate to high ability to focus on their breath during the Focus-on-Breath condition, moderate mind wandering during the Focus-on-Breath condition, and moderate calmness during the task. The scores on these measures did not differ across fMRI runs (OBS, NF-1, NF- 2, NF-3, and TRS; Focus-on-Breath: [F_(4, 135)_ = 0.93, *p* = 0.45; R^2^ = 0.31]; mind wandering: [F_(4, 135)_ = 2.03, *p* = 0.09; R^2^ = 0.45]; and current feeling: [F_(4, 135)_ = 0.65, *p* = 0.63; R^2^ = 0.56]). State mindfulness (SMS) increased from pre- to post-NAMT session [F_(1, 36)_ = 5.82, *p* = 0.02; R^2^ = 0.79]. The PROMIS scales are anchored with a mean of 50 for the United States general population, thus the current sample displayed average levels of life satisfaction (M ± SD = 49.05 ± 8.8), slightly lower levels of meaning and purpose (M ± SD = 47.41 ± 7.59), and slightly higher levels of positive affect (M ± SD = 55.42 ± 6.15), as well as lower levels of fatigue (M ± SD = 43.8 ± 10.62), pain behavior (M ± SD = 35.75 ± 10.52), and pain interference (M ± SD = 40.65 ± 7.48). 

### 3.2. Insula Region of Interest (ROI) Results

Table 3 shows the uncorrected correlations between parameter estimate (Focus-on-Breath vs. Describe) in PCC and insula subregions across runs. Overall, INS subregions were positively correlated with PCC. pINS showed moderate to strong correlations with PCC across runs, while aINS and mINS showed moderate correlation with PCC in all runs except for NF-1, where no correlations were found. Notably, only the correlations between INS subregions and PCC in OBS, as well as pINS and PCC in NF-2 and NF-3 survived the multiple comparison threshold (*p* = 0.05/15 = 0.003).

**Figure A2:**
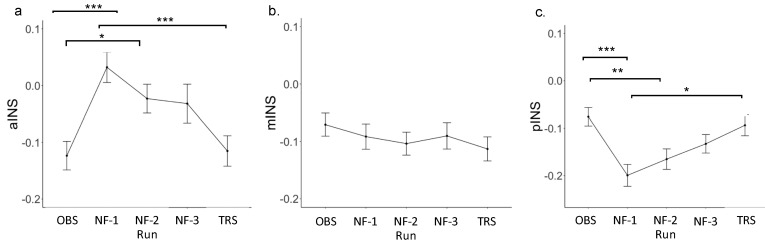
(**a**–**c**), parameter estimate (Focus-on-Breath vs. Rest) across runs for insula subregions. Abbreviations: OBS, Observe; NF, neurofeedback run; TRS, Transfer * *p* < 0.05. ** *p* < 0.01. *** *p* < 0.001.

LME analyses revealed the main effect of run for parameter estimate (Focus-on-Breath vs. Describe) in aINS [*F*_(4, 143)_ = 4.98, *p* < 0.001; R^2^ = 0.39] and pINS [*F*_(4, 143)_ = 11.80, *p* < 0.001; R^2^ = 0.33], but not mINS [*F*_(4, 143)_ = 1.56, *p* = 0.18; R^2^ = 0.34] (Figure 2; Table 4). Post hoc analyses performed for aINS and pINS subregions revealed significant differences between neurofeedback and non-neurofeedback runs. Specifically, parameter estimate (Focus-on-Breath vs. Describe) was greater in neurofeedback runs than OBS in aINS (*p* < 0.01 for NF-1 and NF-2, *p* < 0.05 for NF-3), but was lower in neurofeedback runs than OBS (all *ps* < 0.001) and TRS (*p* < 0.01 for NF-1 and NF-3, *p* < 0.05 for NF-2) in pINS (Table 5). Additional analyses in parameter estimate (Focus-on-Breath vs. Rest) confirmed activation in aINS and deactivation in pINS during neurofeedback runs. For parameter estimate (Focus-on-Breath vs. Rest) across each run for insula subregions, the LME results and post hoc tests are summarized in Appendix B Table A3 and Table A4, respectively. Line graphs are displayed in Appendix B Figure A2.

We also tested parameter estimate (Focus-on-Breath vs. Describe) in aINS, mINS, and pINS across experimental runs, separately (Figure 2). In aINS, parameter estimate for Focus-on-Breath was significantly lower than that in Describe during OBS and TRS [OBS: *t*_(36)_ = −5.21, *p* < 0.001, Cohen’s *d* = −0.86; TRS: *t*_(36)_ = −2.98, *p* < 0.01, Cohen’s *d* = −0.49], but not NF-1, NF-2, and NF-3 runs (all *ps* > 0.10), in mINS during NF-1 and NF-3 [NF-1: *t*_(36)_ = −2.13, *p* < 0.05, Cohen’s *d* = −0.35; NF-3: *t*_(36)_ = −2.50, *p* < 0.05, Cohen’s *d* = −0.41], but not OBS, NF-2, and TRS (all *ps* > 0.05), and in pINS during NF-1, NF-2, and NF-3 [NF-1: *t*_(36)_ = −7.23, *p* < 0.001, Cohen’s *d* = −1.19; NF- 2: *t*_(35)_ = −6.14, *p* < 0.001, Cohen’s *d* = −1.01; NF-3: *t*_(36)_ = −5.83, *p* < 0.001, Cohen’s *d* = −0.96], but not OBS and TRS runs (all *ps* > 0.05). 

### 3.3. Self-Reported Questionnaire Responses and Insula ROI Results

**Figure A3:**
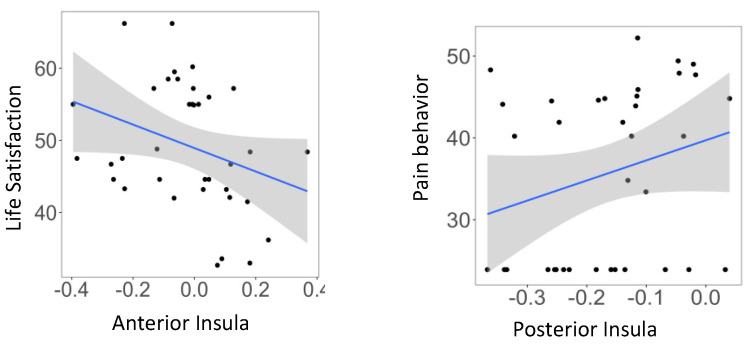
Scatterplot of significant correlations between parameter estimate (Focus-on-Breath vs. Describe) in insula subregions during neurofeedback runs and PROMIS measures.

Relationship between insula subregions and self-reported task ratings and psychological function are reported in Appendix B Table A5 and Figure A3. None of the correlations met Bonferroni correction; *p* = 0.05/9 = 0.005. pINS activity was negatively correlated with mind wandering during OBS, *r* = −0.34, *p* < 0.05, while current feeling (1 = perfectly calm, 10 = very anxious) was negatively correlated with aINS, *r* = −0.43, *p* < 0.01. and mINS, *r* = −0.37, *p* < 0.05 in TRS. During neurofeedback runs, aINS activity was negatively correlated with PROMIS life satisfaction (*r* = −0.37, *p* < 0.05), whereas pINS activity was positively correlated with PROMIS pain behavior (*r* = 0.33, *p* < 0.05).

## 4. Discussion

Based on our previous findings that established the successful downregulation of PCC as a function of rtfMRI-nf-augmented mindfulness training, as well as the co-modulation of the posterior INS [49], the present study sought to directly examine the role of INS in mindfulness in healthy adolescents during the PCC-targeted rtfMRI-nf. Given that different subregions of INS specialize in integrating across the body, cognitive, affective, and awareness domains, we focused our examination on activity in the anterior (aINS), mid- (mINS), and posterior (pINS) subregions and their associations with self-reported affective and sensory measures. The observed correlations between PCC activity and insula subregions confirmed previously reported whole-brain and functional connectivity findings. Contrary to our hypothesis, relative to self-referential processing (i.e., Describe), MT (i.e., Focus-on-Breath) significantly reduced aINS activity during non-neurofeedback runs (OBS and TRS). However, this deactivation in aINS was not further modulated consequent to rtfMRI-nf. Instead, as hypothesized, aINS showed more activation during MT when PCC neurofeedback was given (NF-1 to NF-3). Second, we observed an opposite effect in pINS, such that no differences between conditions were present during non-neurofeedback runs, while significant deactivations occurred for MT during neurofeedback runs. Finally, mINS activity did not differ between conditions during non-neurofeedback runs but reduced for MT during the first and the last neurofeedback runs. Correlational analyses showed that pINS activity was negatively correlated with mind wandering during OBS, while aINS and mINS were negatively correlated with current feeling (calm vs. anxious) during TRS. During neurofeedback runs, aINS activity was negatively correlated with life satisfaction and pINS activity was positively correlated with pain behavior, while no associations were found for mINS activity. These findings provide direct evidence for the involvement of INS in MT and demonstrate that MT elicited distinct responses across INS subregions that may partially explain the previously reported mixed findings regarding INS activity during MT. 

INS subregions have distinct functions. An activation-likelihood-estimation meta-analysis of 1768 neuroimaging studies revealed that sensorimotor tasks consistently activate mid-posterior INS, whereas social-emotional and cognitive tasks activate aINS [78]. Given that aINS is an important hub for interoceptive awareness, accumulated studies have shown aINS to be sensitive to MT relative to mINS and pINS [79,80,81]. For example, following 7- to 8-week MT, aINS activation was observed in healthy participants while focusing attention on breathing, body, and thoughts [36], in patients with general anxiety disorder performing affect labeling of emotional facial expressions [82], and in elite athletes experiencing an interoceptive challenge [83]. Consistent with prior research, we found that, relative to self-referential processing, mindful attention to breathing led to an increase in aINS activity, however, only during neurofeedback runs.

Recent fMRI studies have suggested aINS to be part of the “salience network”, important in attentional control and detecting behaviorally salient stimuli [84,85,86]. Various factors may contribute to activation in the salience network, including increases in cognitive effort, reward anticipation [87,88], heightened sympathetic arousal [89], and the pressure to perform self-regulation within limited time period [90]. Moreover, aINS is critical in detecting discrepancies between actual and desired states [91,92], as well as integrating the feedback display (external) with brain activity (internal) [93]. Neurofeedback training may enhance these processes because participants must integrate information from the feedback and regulate their breathing in order to obtain the desired state. For example, aINS engagement is observed when participants actively tried to move the neurofeedback signal relative to a passively watching condition [94]. Furthermore, neurofeedback training may enhance self-referential processing due to self-evaluation of one’s performance while attempting to control the neurofeedback bar. Therefore, aINS activity during neurofeedback relative to non-neurofeedback MT runs in the present study may reflect the complex process of integrating state awareness of physical sensations of breath with increased attentional control and self-evaluation. A modest trend toward greater deactivation across neurofeedback runs potentially suggests habituation to the neurofeedback signal bar. Together with the minimal difference in aINS activity between OBS and TRS runs, it is possible that aINS activity during neurofeedback training in part reflects the introduced cognitive load of the feedback signal. 

aINS, particularly its dorsal region, has long been linked to subjective well-being, a multidimensional construct that involves both cognitive and affective evaluation of life satisfaction [95,96,97,98,99]. Associations have been found between well-being and INS gray matter volume [100], as well as functional connectivity of the dorsal aINS [98]. Remarkably, aINS activation has been reported to be positively correlated with momentary happiness ratings [95]. This is consistent with our findings which showed a relationship between higher activity in aINS and mINS feeling more calm during the task, as well as between higher activity in pINS and less mind wandering. 

It is well known that MT increases subjective well-being by promoting greater awareness of the present moment [101]. Therefore, greater interference of external stimuli (i.e., attentional and evaluative engagement with the neurofeedback signal bar) during MT may indicate overall propensity toward less present moment awareness in the presence of salient and self-relevant stimuli. This may not only further explain the difference in aINS activity between neurofeedback and non-neurofeedback runs, but also the association between mindful awareness and psychological well-being. 

pINS is considered the primary interoceptive cortex and plays a crucial role in integrating somatic processing and pain perception [85,102,103,104]. Further, pINS has been shown to play a role in momentary self-referential processing [105]. Previous literature is scant on pINS activity in response to MT. In contrast to one study that reported greater pINS activation during an interoceptive breath-focused task in participants who completed MBSR [102], we found that augmenting MT with neurofeedback decreased pINS activation. Deactivation in pINS during neurofeedback runs might be a result of decreased momentary self-referential processing given that participants were engaged in monitoring the feedback signal and with the physical sensations of their breath. This assumption was further supported by the fact that pINS activity was increased during the TRS where the neurofeedback signal (and thus, attentional engagement with the signal bar) was removed, albeit to a lesser extent than during the OBS run. Consistent with past studies showing increased INS activity during processing of painful stimuli [106,107,108], we found that adolescents who reported more pain behaviors also showed increased pINS activity during MT relative to self-referential processing in neurofeedback runs. pINS activity during NAMT may be related to the individual’s levels of sensory processing of pain (e.g., individuals who have greater sensory awareness of pain may have poor inhibited sensory processing of pain), which may lead to the relatively increased pINS activity during MT compared to self-referential processing; however, the mechanism underlying this is unclear. These findings are further linking INS activity to attentional deployment, engagement with physical sensations, and self-referential processing. 

Although mINS activity reduced during NF-1 and NF-3 of MT relative to self-referential processing, it did not differ significantly across experimental runs, nor was it related to any PROMIS pediatric measures. The lack of consistent change in mINS activity during neurofeedback runs could potentially be explained by its unique anatomical position, a connecting structure between aINS and pINS. Thus, mINS activity might be affected by both aINS activation and pINS deactivation during neurofeedback runs [85]. Furthermore, the absence of change in mINS activity across all experimental runs during MT could be due to its main role being integrating interoception with motivated behavior, or selecting action-outcome behaviors [109]. As a result, the current study might fail to engage mINS because it did not provide motivated signals or require action selection. Further research that aims to determine the role of mINS in MT might consider these factors in the experimental design.

INS is involved in a wide variety of functions ranging from sensory and affective processing to high-level cognition [27,85], which may be implicated in the rich cortical connections of INS. For example, aINS has connections with the cingulate cortex, frontal, orbitofrontal, anterior temporal and limbic areas [85,110,111,112,113], affording its role underlying various cognitive and affective functions; as well as the pregenual anterior cingulate and the anterior mid-cingulate that are related with emotional and pain processing [114,115]. mINS mainly projects to the mid-cingulate cortex [116,117], a region important for integrating bodily sensations and exteroceptive sensory afferents [118], supporting its main role as discussed above; while pINS is primarily connected to regions for sensorimotor processing such as posterior temporal, parietal, and sensorimotor areas. Recent rodent model suggests that pINS is also involved in top-down modulation of behavior upon the detection of internal aversive state [119]. 

Given that attention control and acceptance are the two core components of mindfulness [120,121,122], INS function is particularly relevant for MT due to its involvement in interoception [30], self-awareness [123], attention control, and emotion regulation. Attention control enables attending to the moment-to-moment experience (i.e., breathing), which, in turn, cultivating interoceptive awareness and provides an integrated representation of the present moment [93]. In addition, achieving a non-judgmental attitude toward the present experience requires both cognitive reappraisal and emotion regulation [124]. Thus, INS is integral to mindfulness in that it senses, interprets, integrates, and regulates internal and external inputs [125]. Finally, INS-dependent interoceptive regulation is particularly important during adolescence when self-regulatory abilities rapidly develop [126]. Atypical interoception may contribute to onset of psychopathology and decreased socio-emotional competence in late adulthood [127]. Heightened interoceptive reactivity to pleasant stimuli accompanied by increased pINS activity in adolescents relative to young and mature adults may explain developmentally appropriate increased levels of risky decision making during this period of life [128,129,130]. Taken together, findings from our studies and others suggest that MT may influence INS function and interoceptive processing in clinically relevant ways. 

### Limitations

Several limitations should be mentioned for this study. First, although this study was well powered to detect differences in activation across experimental conditions in a single population, the sample size of the present study remains relatively small (*n* = 37). Second, the pain behavior rating is of low variability in nearly half of this healthy sample (*n* = 15 rated 23.9). Future studies would benefit from more diverse samples across the gender, age, race/ethnicity, and clinical domains. Importantly, clinical samples will give evidence to whether INS-level changes translate into modifications in symptom measures. Third, the present study employed rtfMRI-nf targeting PCC and not INS directly. Future work employing rtfMRI-nf targeting INS might provide a more accurate investigation of MT on INS activity, thus to directly evaluate the success of modulating INS activity with neurofeedback and the downstream effects of NAMT targeting INS. Fourth, we did not employ a sham condition against which the effects of PCC-targeted rtfMRI-nf on INS activity could be evaluated. Furthermore, although we provided the associations between PCC and INS subregions, future work should examine changes in functional connectivity between the INS subregions and PCC as a function of rtfMRI-nf using resting state data to provide more details regarding this relationship. Lastly, it is worth noting that the self-referential task might elicit emotional responses that further induce bodily change because participants were instructed to determine whether the adjectives described themselves or not [131]. Future studies could improve the control task to provide a more stable reference by avoiding instructions that elicit potential bodily changes.

## 5. Conclusions

This is the first study to examine activity in INS subregions during PCC-targeted rtfMRI-nf MT in healthy adolescents. These findings add to the existing literature for the integral role of INS in MT. The data also showed a relation between INS activity during NAMT and self-reported cognitive/affective/sensory processing. The divergent effect of PCC rtfMRI-nf on anterior vs. posterior INS subregions during mindfulness practice relative to self-referential processing may support previous findings whereby aINS is involved in the experience of cognitive-affective states, while pINS plays a more prominent role in somatosensory processes. Future studies are needed to directly examine how distinct mindfulness practices modulate INS along the proposed subregion specializations given that we focused only on focused-attention MT. Finally, studies with larger and clinical samples will determine whether MT impacts INS activity and interoceptive processes to improve clinical outcomes.

## Figures and Tables

**Table 1:** Sample demographic information.

Demographic	%
Race	
White	71
Black	2
Asian	5
American Indian/Alaska Native	11
Biracial/Multiracial	11
Education	
7th grade	18
8th grade	38
9th grade	18
10th grade	16
11th grade	7
13th grade	3
Family Income	
$0–$49,999	7
$50,000–$99,999	38
$100,000–$149,999	21
$150,000–$199,999	17
>$200,000	17

**Table 2:** Unadjusted means, standard deviations, effect sizes, and main analyses of task ratings and symptom measures.

Task Ratings	Mean	SD	Estimate	SE	t	*p*	Cohen’s d
Focus-on-Breath							
OBS	7.09	1.54					
NF-1	6.56	1.67	−0.51	0.36	−1.42	0.16	−0.25
NF-2	6.45	1.70	−0.63	0.36	−1.72	0.09	−0.30
NF-3	6.70	2.04	−0.37	0.35	−1.05	0.30	−0.18
TRS	6.53	1.61	−0.50	0.35	−1.42	0.16	−0.25
Mind Wander							
OBS	5.23	2.12					
NF-1	4.85	1.86	−0.47	0.36	−1.31	0.19	−0.23
NF-2	5.21	2.09	−0.11	0.36	−0.31	0.76	−0.05
NF-3	5.83	1.83	0.10	0.35	0.29	0.77	0.05
TRS	5.75	1.84	0.45	0.36	1.26	0.21	0.22
Current Feeling							
OBS	2.89	1.53					
NF-1	3.41	1.76	0.38	0.29	1.30	0.20	0.22
NF-2	3.06	1.69	0.15	0.29	0.51	0.61	0.09
NF-3	3.03	1.78	0.05	0.29	0.16	0.87	0.03
TRS	3.06	2.04	0.10	0.29	0.34	0.74	0.06
Measure	Mean	SD	Estimate	SE	t	*p*	Cohen’s d
State Mindfulness Scale (SMS)							
T1	71.22	14.09					
T2	74.68	12.69	3.46	1.43	2.41	<0.05	0.53

Note. Task ratings were answered following the completion of each run. Questions included “ How easy did you find it to focus on your breath?” (1 = not easy at all; 10 = very easy); “How much did your mind wander while you were asked to focus on your breath?” (1 = not at all; 10 = all the time); “How do you feel right now?” (1 = perfectly calm; 10 = very anxious). Abbreviations: OBS, Observe; NF, Neurofeedback; TR, Transfer; T1, Pre-NAMT; T2, Post-NAMT.

**Table 3:** Uncorrected correlations between parameter estimate (Focus-on-Breath vs. Describe) in PCC and insula subregions across runs.

INS Subregions	Run	PCC_OBS	PCC_NF-1	PCC_NF-2	PCC_NF-3	PCC_TRS
aINS	OBS	0.65 ***	-	-	-	-
	NF-1	-	0.12	-	-	-
	NF-2	-	-	0.35 *	-	-
	NF-3	-	-	-	0.37 *	-
	TRS	-	-	-	-	0.34 *
mINS	OBS	0.69 ***	-	-	-	-
	NF-1	-	0.09	-	-	-
	NF-2	-	-	0.36 *	-	-
	NF-3	-	-	-	0.42 **	-
	TRS	-	-	-	-	0.35 *
pINS	OBS	0.71 ***	-	-	-	-
	NF-1	-	0.45 **	-	-	-
	NF-2	-	-	0.56 ***	-	-
	NF-3	-	-	-	0.53 ***	-
	TRS	-	-	-	-	0.42 **

Abbreviations: NF, Neurofeedback; OBS, Observe; TR, Transfer. * *p* < 0.05. ** *p* < 0.01. *** *p* < 0.001.

**Table 4:** Unadjusted means, standard deviations, effect sizes, and main analyses of fMRI coefficient for the Focus-on-Breath vs. Describe contrast in subregions of insula across runs.

Run	Mean	SD	Estimate	SE	t	*p*	Cohen’s d
Anterior insular cortex (aINS)							
OBS	−0.16	0.19					
NF-1	−0.01	0.18	0.16	0.04	3.81	<0.001	0.63
NF-2	−0.02	0.21	0.14	0.04	3.26	<0.01	0.54
NF-3	−0.05	0.28	0.11	0.04	2.75	<0.01	0.46
TRS	−0.12	0.24	0.05	0.04	1.16	<0.001	0.19
Mid-insular cortex (mINS)							
OBS	0	0.18					
NF-1	−0.06	0.17	−0.06	0.03	−1.70	0.09	−0.28
NF-2	−0.05	0.15	−0.05	0.03	−1.47	0.15	−0.24
NF-3	−0.08	0.2	−0.08	0.03	−2.42	0.05	−0.40
TRS	−0.06	0.19	−0.06	0.03	−1.63	0.11	−0.27
Posterior insular cortex (pINS)							
OBS	0.01	0.17					
NF-1	−0.17	0.14	−0.18	0.03	−5.25	<0.001	−0.88
NF-2	−0.16	0.15	−0.17	0.03	−4.91	<0.001	−0.82
NF-3	−0.17	0.18	−0.18	0.03	−5.27	<0.001	−0.88
TRS	−0.05	0.17	−0.06	0.03	−1.72	0.09	−0.29

Abbreviations: OBS, Observe; NF, Neurofeedback; TRS, Transfer.

**Table 5:** Post hoc comparisons for parameter estimates (Focus-on-Breath vs. Describe) in INS subregions across runs.

Run	Estimate	Std. Error	z Statistic	*p* Value
Anterior insular cortex (aINS)				
NF-1:OBS	0.16	0.04	3.81	<0.01
NF-2:OBS	0.14	0.04	3.26	<0.05
NF-3:OBS	0.11	0.04	2.75	<0.05
TR:OBS	0.05	0.04	1.16	0.78
NF-2:NF-1	−0.02	0.04	−0.52	0.98
NF-3:NF-1	−0.04	0.04	−1.06	0.83
TR:NF-1	−0.11	0.04	−2.66	0.06
NF-3:NF-2	−0.02	0.04	−0.53	0.98
TR:NF-2	−0.09	0.04	−2.11	0.22
TR:NF-3	−0.07	0.04	−1.60	0.50
Mid-insular cortex (mINS)				
NF-1:OBS	−0.06	0.03	−1.70	0.43
NF-2:OBS	−0.05	0.03	−1.47	0.59
NF-3:OBS	−0.08	0.03	−2.42	0.11
TR:OBS	−0.06	0.03	−1.63	0.48
NF-2:NF-1	0.01	0.03	0.22	1.00
NF-3:NF-1	−0.02	0.03	−0.72	0.95
TR:NF-1	0.00	0.03	0.07	1.00
NF-3:NF-2	−0.03	0.03	−0.93	0.89
TR:NF-2	−0.01	0.03	−0.15	1.00
TR:NF-3	0.03	0.03	0.79	0.93
Posterior insular cortex (pINS)				
NF-1:OBS	−0.18	0.03	−5.25	<0.001
NF-2:OBS	−0.17	0.03	−4.91	<0.001
NF-3:OBS	−0.18	0.03	−5.27	<0.001
TR:OBS	−0.06	0.03	−1.72	0.42
NF-2:NF-1	0.01	0.03	0.30	1.00
NF-3:NF-1	0.00	0.03	−0.02	1.00
TR:NF-1	0.12	0.03	3.52	<0.01
NF-3:NF-2	−0.01	0.03	−0.32	1.00
TR:NF-2	0.11	0.03	3.20	<0.05
TR:NF-3	0.12	0.03	3.55	<0.01

Abbreviations: NF, Neurofeedback; OBS, Observe; TR, Transfer.

## Data Availability

Data will be available upon request from the corresponding author.

## Appendix A

### Appendix A.1. Neurofeedback-Augmented Mindfulness Training Task (NAMT)

Prior to the rtfMRI-nf session, adolescents underwent brief mindfulness training (MT). Participants were first given a brief psychoeducational introduction into mindfulness, including that (1) mindfulness refers to paying attention to thoughts, feelings, and physical sensations in the present moment without any judgment, and (2) mindfulness can reduce stress and increase attention. Next, participants were guided through a traditional mindfulness practice focused on the breath [43,48], that is: “*Please pay attention to the physical sensations of your breath where you most strongly feel it. Follow the natural and spontaneous movement of the breath, not trying to change it any way. Just pay attention to it. If you find that your attention wanders to something else, gently but firmly bring it back to the physical sensations of the breath*.” Difficulty of performing the task and how mindful they currently feel of their body and mind were assessed. Following practice, adolescents were provided with an opportunity to ask clarification questions. Next, adolescents went into the mock scanner and completed the same mindfulness practice and assessment with MRI noises in the background. Adolescents were also given instructions and feedback around minimizing motion while in the scanner. Finally, adolescents were given instructions for the neuroimaging session. Training was manualized to ensure fidelity across participants. MT was delivered by a trained research assistant under the supervision of a licensed clinical psychologist. Training sessions were audio recorded and up to 20% sessions were randomly selected for fidelity ratings by research staff.

The neuroimaging session included 8 runs (Figure 1a), including an anatomical scan, Resting State scan 1 (Rest-1), Observe (OBS), three neurofeedback runs (NF-1, NF-2, NF-3), Transfer run (TRS), and Resting State scan 2 (Rest-2). During Rest-1 and Rest-2 (6 min each), participants were instructed to clear their mind and not think about anything while fixating upon a fixation cross. OBS, NF-1, NF-2, NF-3, and TRS runs each lasted 6 min and 56 s. Runs started with a 66 s rest block, followed by alternating Describe (Active Control condition without neurofeedback; 20 s), Focus-on-Breath (MT condition with PCC neurofeedback; 70 s), and Rest (Baseline condition; 30 s) blocks. OBS and TRS runs did not involve neurofeedback (no bar displayed) during the Focus-on-Breath condition. The initial long Rest block was required for obtaining enough samples for real-time noise regression analysis [132].

During the Focus-on-Breath condition [48], adolescents were instructed to pay attention to the physical sensations of their breath, not trying to change their breathing in any way, and if their attention were to wander to something else, to gently bring their attention back to their breath [43]. To aid in MT, numerous useful strategies were provided prior to scanning, including “Notice the feeling of your belly rising when you breath in, and gently falling when you breath out”; “Notice if it enters and leaves through your nose or your mouth.” In the Describe condition, adolescents were presented with various adjectives, which they had to mentally categorize as descriptive or not descriptive of them for the entire duration the word was displayed on the screen [61]. The Describe condition is designed to elicit self-referential thinking, and therefore is thought to be a better comparator to Focus-on-Breath than Rest [43]. During the Rest condition, adolescents were presented with the cue “Rest” and asked to relax while looking at the display screen.

During neurofeedback runs, adolescents were told that they would see a bar displayed on the screen, representing the relative brain activity in a particular brain region in real time (Figure 1b). The instructions further indicated that the bar may change with the experience of focusing on the breath (i.e., the bar may go blue if they are fully concentrating on their breath, and red if their mind wanders elsewhere). The green bar represented the target to attain, and adolescents’ goal was to try and see how much they could make the bar change to blue to match the green bar. The target levels were −0.5%, −0.75%, and −1.0% (percent signal change is relative to the preceding rest block) for the NF-1, NF-2, and NF-3 runs, respectively. Adolescents were told that there might be a 5–6 s delay between their experience and the change in the blue bar. To assess for aspects of feasibility and tolerability, adolescents answered the following questions via a response box and visual analog scale after reach run: (1) How well were you able to follow instructions on the screen? (2) How easy did you find it to focus on your breath? (3) How much did your mind wander while you were asked to focus on your breath? (4) How easy did you find it to mentally decide whether or not the words described you? (5) How easy did you find it to clear your mind while resting? (6) How do you feel right now (1 = perfectly calm, 10 = very anxious)? Two additional questions followed the neurofeedback runs only: (1) How well did the blue bar correspond with your experience of focusing on your breath? (2) How well did the red bar correspond with the experience of your mind wandering elsewhere?

### Appendix A.2. Real-Time fMRI Processing

The region of interest (ROI) and rtfMRI-nf target location (spherical ROI, 7mm radius, [MNI coordinates: x = −5, y = −55, z = 23]; Figure 1a) were selected based on a meta-analysis investigating functional neuroimaging studies of the DMN [133], mindfulness meditation studies, including neurofeedback [43,48], and conducted pilot testing.

We employed an advanced fMRI real-time processing (RTP) protocol which included slice-timing correction, motion correction, spatial smoothing with 6 mm-FWHM Gaussian kernel within the brain mask, scaling to a percent change relative to the average for the first 19 TRs (in the initial rest period), and regressing out noise components [72,132]. The noise regressors were six motion parameters, eight RETROICOR [67] regressors (four cardiac and four respiration), white matter mean signal, ventricle mean signal, and Legendre polynomial models of slow signal fluctuation. This comprehensive noise reduction was performed in real-time (less than 400 ms [132]). This fMRI RTP system operates real-time motion tracking, alignment, and motion parameter regression, thus allowing for suppression of head motion effects, and importantly, providing physiological noise correction (RETROICOR) in real time before the PCC-based neurofeedback signal computation and visual presentation to the adolescent [132,134,135]. This ensured that the PCC neurofeedback signal reflected the largest possible extent the underlying neuronal activity and does not reflect head motion, heart rate, and/or respiratory motions, all of which overlap with DMN neural activity [136,137]. After real-time noise regression, the fMRI RTP system exports the mean value of the noise-reduced signals for the PCC ROI for each acquired data volume.

The PCC ROI (i.e., neurofeedback target signal) in the MNI space was warped into the individual brain space using the Advanced Normalization Tools (ANTs) software [69] (http://stnava.github.io/ANTs/). The neurofeedback stimulus was delivered via custom-developed software using PsychoPy [138]. The neurofeedback value was a signal change relative to the baseline obtained by averaging the preceding 30 s long Rest condition. The two initial volumes in the Rest condition were excluded from the baseline calculation to avoid the delayed hemodynamic response effect of the preceding Describe block. The neurofeedback started from the third volume in the Focus block to wait for a hemodynamic response delay. The bar height was updated at every TR as a moving average of the current and up to the two available preceding values to reduce the bar fluctuation [139]. The consensus on the reporting and experimental design of clinical and cognitive-behavioral neurofeedback studies (CRED-nf checklist) is shown in Appendix B Table A1 [62].

## Appendix B

**Table A1:** Consensus on the reporting and experimental design of clinical and cognitive-behavioral neurofeedback studies (CRED-nf) best practices checklist 2020.

Domain	Item #	Checklist Item	Reported on Page #
Pre-experiment			
	1a	Pre-register experimental protocol and planned analyses	n/a
	1b	Justify sample size	4
Control groups			
	2a	Employ control group(s) or control condition(s)	5
	2b	When leveraging experimental designs where a double-blind is possible, use a double-blind	n/a
	2c	Blind those who rate the outcomes, and when possible, the statisticians involved	n/a
	2d	Examine to what extent participants and experimenters remain blinded	n/a
	2e	In clinical efficacy studies, employ a standard-of-care intervention group as a benchmark for improvement	n/a
Control measures			
	3a	Collect data on psychosocial factors	5–6
	3b	Report whether participants were provided with a strategy	5
	3c	Report the strategies participants used	n/a
	3d	Report methods used for online-data processing and artifact correction	6–7
	3e	Report condition and group effects for artifacts	n/a
Feedback specifications			
	4a	Report how the online-feature extraction was defined	6
	4b	Report and justify the reinforcement schedule	n/a
	4c	Report the feedback modality and content	4–5
	4d	Collect and report all brain activity variable(s) and/or contrasts used for feedback, as displayed to experimental participants	4–5
	4e	Report the hardware and software used	6–9
Outcome measures			
Brain	5a	Report neurofeedback regulation success based on the feedback signal	n/a
	5b	Plot within-session and between-session regulation blocks of feedback variable(s), as well as pre-to-post resting baselines or contrasts	n/a
	5c	Statistically compare the experimental condition/group to the control condition(s)/group(s) (not only each group to baseline measures)	n/a
Behavior	6a	Include measures of clinical or behavioral significance, defined a priori, and describe whether they were reached	n/a
	6b	Run correlational analyses between regulation success and behavioral outcomes	n/a
Data storage			
	7a	Upload all materials, analysis scripts, code, and raw data used for analyses, as well as final values, to an open access data repository, when feasible	n/a

Note: Darker shaded boxes represent *Essential* checklist items; lightly shaded boxes represent Encouraged checklist items. We recommend using this checklist in conjunction with the standardized CRED-nf online tool (rtfin.org/CREDnf) and the CRED-nf article, which explains the motivation behind this checklist and provides details regarding many of the checklist items.

**Table A2:** Peak coordinates of the clusters from group fMRI analysis for the mean Focus-on-Breath vs. Describe contrast. Insular regions defined by the Brainnetome atlas [73].

Gyrus	ROI	Label ID.L	Label ID.R	Anatomical and Modified Cyto-Architectonic Descriptions	Left Hemisphere MNI Coordinates	Right Hemisphere MNI Coordinates
Insular Gyrus	Anterior Insula	165	166	*vIa, ventral agranular insula*	−32, 14, −13	33, 14, −13
		167	168	*dIa, dorsal agranular insula*	−34, 18, 1	36, 18, 1
	Mid-Insula	169	170	*vId/vIg, ventral dysgranular and granular insula*	−38, −4, −9	39, −2, −9
		173	174	*dId, dorsal dysgranular insula*	−38, 5, 5	38, 5, 5
	Posterior Insula	163	164	*G, hypergranular insula*	−36, −20, 10	37, −18, 8
		171	172	*dIg, dorsal granular insula*	−38, −8, 8	39, −7, 8

Note. Anterior insula was obtained by combining left and right hemisphere, dorsal and ventral agranular insula. Mid-insula was obtained by combining left and right hemisphere, ventral dysgranular and granular insula, and dorsal dysgranular insula. Posterior insula was obtained by combining left and right hemisphere, hypergranular insula, and dorsal granular insula. See Figure 2a.

**Table A3:** Unadjusted means, standard deviations, effect sizes, and main analyses of parameter estimate (Focus-on-Breath vs. Rest) in insula subregions across runs.

Run	Mean	SD	Estimate	SE	t	*p*	Cohen’s d
Anterior insula							
OBS	−0.12	0.17					
NF-1	0.03	0.18	0.16	0.04	4.40	<0.001	0.74
NF-2	−0.02	0.17	0.10	0.04	2.74	<0.01	0.46
NF-3	−0.03	0.23	0.09	0.04	2.59	<0.05	0.43
TRS	−0.12	0.18	0.01	0.04	0.23	0.81	0.04
Mid-insula							
OBS	−0.07	0.14					
NF-1	−0.09	0.15	−0.02	0.03	−0.75	0.45	−0.13
NF-2	−0.10	0.14	−0.03	0.03	−1.22	0.23	−0.20
NF-3	−0.09	0.16	−0.02	0.03	−0.71	0.48	−0.12
TRS	−0.11	0.14	−0.04	0.03	−1.53	0.13	−0.26
Posterior insula							
OBS	−0.08	0.13					
NF-1	−0.20	0.16	0.03	0.03	1.11	0.27	0.19
NF-2	−0.17	0.15	0.07	0.03	2.25	<0.05	0.38
NF-3	−0.13	0.13	0.12	0.03	4.16	<0.001	0.70
TRS	−0.09	0.15	0.11	0.03	3.56	<0.001	0.60

Abbreviations: OBS, Observe; NF, neurofeedback run; TRS, Transfer.

**Table A4:** Post hoc comparisons for parameter estimate (Focus-on-Breath vs. Rest) in insula subregions across runs.

Run	Estimate	Std. Error	z Statistic	*p* Value
Anterior insular cortex (aINS)				
NF-1:OBS	0.16	0.04	4.40	<0.001
NF-2:OBS	0.10	0.04	2.74	0.05
NF-3:OBS	0.09	0.04	2.59	0.07
TR:OBS	0.01	0.04	0.23	1.00
NF-2:NF-1	−0.06	0.04	−1.63	0.48
NF-3:NF-1	−0.06	0.04	−1.81	0.37
TR:NF-1	−0.15	0.04	−4.17	<0.001
NF-3:NF-2	−0.01	0.04	−0.17	1.00
TR:NF-2	−0.09	0.04	−2.50	0.09
TR:NF-3	−0.08	0.04	−2.36	0.13
Mid-insular cortex (mINS)				
NF-1:OBS	−0.02	0.03	−0.75	0.94
NF-2:OBS	−0.03	0.03	−1.22	0.74
NF-3:OBS	−0.02	0.03	−0.71	0.96
TR:OBS	−0.04	0.03	−1.53	0.54
NF-2:NF-1	−0.01	0.03	−0.47	0.99
NF-3:NF-1	0.00	0.03	0.04	1.00
TR:NF-1	−0.02	0.03	−0.78	0.94
NF-3:NF-2	0.01	0.03	0.52	0.99
TR:NF-2	−0.01	0.03	−0.30	1.00
TR:NF-3	−0.02	0.03	−0.82	0.92
Posterior insular cortex (pINS)				
NF-1:OBS	−0.12	0.03	−4.16	<0.001
NF-2:OBS	−0.09	0.03	−3.02	<0.05
NF-3:OBS	−0.06	0.03	−1.92	0.31
TR:OBS	−0.02	0.03	−0.60	0.98
NF-2:NF-1	0.03	0.03	1.11	0.80
NF-3:NF-1	0.07	0.03	2.25	0.16
TR:NF-1	0.11	0.03	3.56	<0.01
NF-3:NF-2	0.03	0.03	1.12	0.80
TR:NF-2	0.07	0.03	2.43	0.11
TR:NF-3	0.04	0.03	1.32	0.68

Abbreviations: NF, Neurofeedback; OBS, Observe; TR, Transfer.

**Table A5:** Correlations between parameter estimate (Focus-on-Breath vs. Describe) in insula subregions during neu-rofeedback runs and variables of interest.

	Neurofeedback Runs			Observe Run			Transfer Run		
	aINS	mINS	pINS	aINS	mINS	pINS	aINS	mINS	pINS
PROMIS Positive Affect	−0.2	−0.15	−0.1	−0.24	−0.16	−0.19	0.08	0.15	0.16
PROMIS Meaning and Purpose	−0.23	−0.24	−0.06	−0.23	−0.09	−0.19	0.07	0.09	0.22
PROMIS Life Satisfaction	−0.37 *	−0.32	−0.19	−0.21	−0.2	−0.17	0.06	0.00	0.11
PROMIS Pain Interference	0.2	0.18	0.29	0.12	0.03	0.15	0.15	0.09	0.12
PROMIS Pain Behavior	0.18	0.32	0.33 *	−0.09	−0.02	−0.04	0.13	0.20	0.18
PROMIS Fatigue	0.28	0.3	0.23	0.27	0.21	0.29	0.06	0.05	0.10
Task Ratings Current Feeling	−0.13	−0.22	−0.25	0.01	−0.25	−0.18	−0.43 **	−0.37 *	−0.19
Task Ratings Mind Wander	−0.31	−0.36	−0.24	−0.11	−0.24	−0.34 *	−0.20	−0.28	−0.22
Task Ratings Focus-on-Breath	−0.03	−0.14	−0.18	−0.14	−0.17	−0.04	0.11	0.26	0.16

Note. aINS: anterior insular cortex, mINS: mid-insular cortex, pINS: posterior insular cortex. PROMIS = Patient-Reported Outcomes Measurement Information System. Task rating scores were averaged across three neurofeedback runs.* *p* < 0.05. ** *p* < 0.01.
